# Field-Control, Phase-Transitions, and Life’s Emergence

**DOI:** 10.3389/fphys.2012.00366

**Published:** 2012-10-05

**Authors:** Gargi Mitra-Delmotte, A. N. Mitra

**Affiliations:** ^1^Independent Researcher, St.DenisReunion, France; ^2^Department of Physics, Delhi UniversityNew Delhi, India

**Keywords:** field-controlled colloids, proto-metabolic cycle, slow driving, long-range correlation, organic “takeover,” phase-transition, feedback

## Abstract

Instances of critical-like characteristics in living systems at each organizational level (bio-molecules to ecosystems) as well as the spontaneous emergence of computation (Langton), do suggest the relevance of self-organized criticality (SOC). But extrapolating complex bio-systems to life’s origins, brings up a paradox: how could simple organics – lacking the “soft-matter” response properties of today’s complex bio-molecules – have dissipated energy from primordial reactions (eventually reducing CO_2_) in a controlled manner for their “ordering”? Nevertheless, a causal link of life’s macroscopic irreversible dynamics to the microscopic reversible laws of statistical mechanics is indicated via the “functional-takeover” of a soft magnetic scaffold by organics (c.f. Cairns-Smith’s “crystal-scaffold”). A field-controlled structure offers a mechanism for boot-strapping – bottom-up assembly with top-down control: its super-paramagnetic colloidal components obey reversible dynamics, but its dissipation of magnetic (H)-field energy for aggregation breaks time-reversal symmetry. The responsive adjustments of the controlled (host) mineral system to environmental changes would bring about mutual coupling between random organic sets supported by it; here the generation of long-range correlations within organic (guest) networks could include SOC-like mechanisms. And, such cooperative adjustments enable the *selection* of the *functional* configuration by altering the inorganic dipolar network’s capacity to assist a spontaneous process. A non-equilibrium dynamics could now drive the kinetically oriented system (trimming the phase-space via sterically coupled organics) toward a series of phase-transitions with appropriate organic replacements “taking-over” its functions. Where available, experiments are cited in support of these speculations and for designing appropriate tests.

## Introduction

The implications of minerals in life’s emergence were first envisaged by Goldschmidt (1952) and Bernal (1949); these included concentration (adsorption) and catalysis, besides chirality of organics via association with crystal-surfaces. This motivated many works (see Carter, 1978; Siegel and Siegel, 1981; Ferris, 1999; Lahav, 1999; Jacoby, 2002; Arrhenius, 2003; Schoonen et al., 2004; Lambert, 2008; Hazen and Sverjensky, 2010, and references therein), and inspired scenarios exploring the resemblance of ancient enzyme-clusters to mineral ones in metabolism-first approaches to life’s origins [Wächtershäuser, 1988; Russell et al., 1989, 1994 (see The Mound Scenario); Cody et al., 2000; McGlynn et al., 2009]. Hazen (2006) reviews the role of mineral surfaces for assistance at two stages of increasing complexity, viz. (1) emergence of bio-molecules, and (2) emergence of macromolecular systems. These in turn cover three aspects: (i) possible enhanced self-assembly of lipids in the presence of minerals (Deamer and Pashley, 1989; Luisi, 1989; Hanczyc et al., 2003; Chen et al., 2004); (ii) polymerization of amino-acids and nucleic acids, (Lahav et al., 1978; Ferris, 1993; Sowerby et al., 1996; Liu and Orgel, 1998; Orgel, 1998; Uchihashi et al., 1999) where Smith (1998) uses channels of zeolites as a packing constraint to help polymerization; and (iii) selective adsorption onto mineral surfaces, of organics (Carter, 1978; Lowenstam and Weiner, 1989; Churchill et al., 2004). The latter include chiral molecules (Lahav, 1999; Jacoby, 2002; Hazen and Sholl, 2003), although Hazen (2006) also mentions other mechanisms for chiral selection like determinate vs. chance local processes. Apart from parity violation in beta-decay; he considers chiral-selective photolysis by circular-polarized synchrotron radiation from neutron stars (Bailey et al., 1998; Podlech, 1999); magnetochiral photochemistry (Rikken and Raupach, 2000); and at smaller scales the amplification of slight chiral excesses via Bose–Einstein condensation (Chela-Flores, 1994), or chiral self-assembly of polymers (Bolli et al., 1997; Lippmann and Dix, 1999; Saghatelian et al., 2001) or simply crystals (Eckert et al., 1993; Lahav and Leiserowitz, 1999).

**Figure 1:**
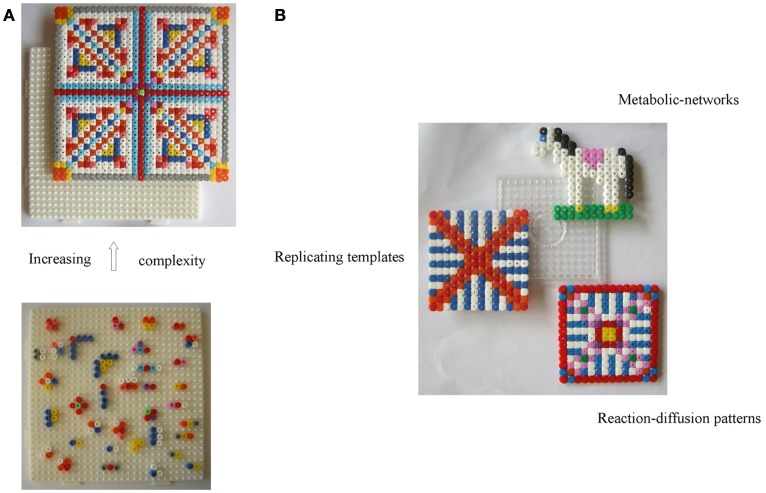
**Toward facilitating the evolution of organic reactions/interactions (guest-level-II) via a controlled inorganic scaffold (host-level-I) *a la* Cairns-Smith. (A)** The probability of forming complex stable dynamical patterns decreases with increasing number of organic molecules. This can be aided via selection by a pre-existing functioning organization – the crystal-scaffold or level-I (represented by a white pin board) acting as “traps” for functioning assembled modules from level-II (represented by a “bottom-up assembly” of colored beads). For, e.g., a variety of recognition-like interactions between organic “building blocks” are required (not all are shown) to construct the unit leading up to the fourfold symmetric structure. Shown on top is the new organic organization which has functionally replaced the original crystal one at level-I. **(B)** To make this scenario compatible with soft colloidal dynamics and facilitate the “takeover” of level-I by a hierarchy of functioning modules, we suggest a reversible field-stabilized scaffold with a modular organization – represented by a transparent pin board. A stable inorganic scaffold is also compatible with the simultaneous emergence of (and replacement by) different types of organic spatio-temporal correlations, and as each of these would be dependent on the scaffold, any external tinkering with the latter’s degrees of freedom (d.o.f.s), would also impact the different organic networks and *facilitate their mutual coupling* (see text).

According to Hazen (2006), Cairns-Smith’s (1968) theory is the most extreme form of mineral-based hypotheses positing that clay crystals were the precursors of today’s replicators. As we see it, in this two-level scenario, the *hosting* inorganic layer or the crystal-organization – call it level-I (depicted as a white pin board in Figure 1A), – offers top-down control and assistance for the bottom-up assembly of organic materials into complex patterns building up from *randomly* reacting/interacting entities in the “*guest*” layer – call it level-II (depicted with colored beads, lower Figure 1A). In the latter, chemical reactions lead to building blocks, small polymers, proto-metabolic reactions, etc., while weak physical interactions (e.g., Hunding et al., 2006) lead to small assemblies. Now, level-I’s own crude *functional organization* acts as a selection/“trimming” mechanism for “fishing out” constructs with superior function (information-propagation capacity) from the multitude of species forming at level-II. This leads to a gradual replacement of the inorganic organization by organic modules (colored pattern, upper Figure 1A), whose recruitment by a functional system – aided by complementary interactions – is crucial for their dynamic stability (see Liquid Crystals; Scaffold Paradigm; and Bottom-Up Approaches); conceptually too, this *relates structure of the organic module to its function*. Also, level-II products favoring propagation of template-information (level-I) enable *feedback* between the levels. But, unlike hard crystals, a soft fractal organization seems a more natural origin for bio-complexity. To that end, a colloidal-gel scaffold (see Magnetic Framboids; the Mound Scenario; a Fractal Scaffold) seems promising as a dynamically stable confining medium compatible with the key role of diffusion-controlled reactions in cellular biochemistry (Kopelman, 1989; Konkoli, 2009). A gradual “takeover” by organic modules is also easier to visualize via a dynamic inorganic modular organization, e.g., soft colloids (Russell et al., 1990), provided one can associate them with a crystal-like organization, toward a formal theory.

Now, unlike mineral-based bottom-up approaches adhering to the “metabolism-first” camp, the crystal-scaffold theory proposes a pre-existing template-organization, thus upholding the “genes-first” one. The former tells how *local* mineral-organic interactions can assist guest-level-II reactions, while the latter considers *global* aspects, i.e., bio-like functions linked to a cooperative organization of mineral-hosts. Indeed, these are complementary, and roughly correspond to the two-tier organization of living systems: the control-network-level-I of complex bio-molecules (proteins, nucleic acids, lipids, carbohydrates, etc.) maps to the hosting functional mineral-organization, and the metabolic-network-level-II maps to the (guest) organic reactions/interactions. In this federal-like anatomy of a living system, each tier/subsystem functions independently – albeit constrained by feedback-coupling (c.f. life’s irreducible structure; Polyani, 1968). Now, the second correspondence – between guest reactions/interactions and metabolic-network-level-II – is easier to visualize than the first one (see Liquid Crystals; Scaffold Paradigm; and Bottom-Up Approaches). Indeed, while *macroscopic energy flow* in the metabolic reaction cycles can be mapped to that in similar organic attractors in abiogenesis, we still need a mapping – albeit in terms of inorganic matter – for the control-network (level-I) capable of *microscopic energy transactions*. This can be seen at the level of the *components* undergoing infinitesimal conformational changes to traverse a continuous energy landscape, or even at the global *system* level, where diverse closely spaced states in genotype-space are accessible via environmental fluctuations. Sure enough, open living systems can *harness fluctuations* – at component (for work-cycles) and (evolving) system levels – unlike technological devices, sealing off external noise.

Now, mechanisms consuming free energy in the least time provide a natural basis for energy flows to select (pre-biotic/genetic) amongst dissipative structures (Annila and Salthe, 2012). And selection could have started on “technologically simpler” (Cairns-Smith, 2008) variations in energy channeling mechanisms (c.f. complex organic functional networks building up from scratch): Analogous to interdependent metabolism and replication, in abiogenesis, *a spontaneous process* provides a thermodynamic incentive for sustaining the continuity of an *environment-coupled cooperative network* assisting its occurrence (function). Complex replacements of the network could have arisen via different mechanisms, including self-organized criticality (SOC; see Living Systems; SOC; and Life’s Emergence) among candidates despite its lack of a predictable framework. Note that otherwise in the origins of life its role seems limited if only guest-level-II processes are considered, since proto-metabolic reactions or weak interactions between organics dispersed in random mixtures alone cannot suffice for SOC to be effective. [As for relative orders of magnitude, bond energies involved in covalent bonds vs. those for Van der Waals clusters bear the ratio: several eV vs. a fraction of an eV (Kreuzer, 2005), compared to thermal energy (*k*_B_*T*) of ∼100th of an eV]. In the absence of an instructional principle, a random process of putting together simple organic building blocks (or mineral-particle-bound ones), into an intricate informational system would seem futile in view of the negligible probabilities at each step, for one wonders what interactive mechanisms are needed to ensure that random mixtures be *stable* enough to stay together to facilitate long-range correlations between them. Thus to reconcile the slow evolution of pure thermodynamic processes with the faster one of life’s, a mechanism (e.g., trimming phase-space) is needed to break free from the constraints of thermodynamics, while paying obeisance to it. To that end, inspired by Cairns-Smith’s pre-existing crystal-organization, we look to the signatures of *fields* on some collectively interacting entities at the inorganic-host-level-I that could have conferred on them the capacity to assist in the advanced stages of complexity, viz. emergence of replicators evolving via natural selection (Hazen, 2006). In particular, the advantage of an external H-field-cum-magnetic nano-particles (MNPs), vis-à-vis say an electric field controlled system of particles, relates to the *diamagnetic properties of nano-sized organics*; thus anchoring the latter to mineral colloids responsive to an H-field can be used as an indirect means to exert control on them (there being associated dielectric properties with both mineral and organic colloids).

Among possible scenarios, one may consider its potential to give its responsive nano-scale materials (1) a dynamical basis of orientation in a liquid phase enabling formation of aggregates due to dipolar interactions (Taketomi, 2011), leading to (2) a *response to the external by the generated internal field* in the interacting system (Huke and Lücke, 2004; see Field-Controlled Scaffold Organization), whose global evolution would also depend on the susceptibility of its materials to external factors (such as temperature). This quintessential analog-information system (Palm and Korenivski, 2009) seems plausible as a scaffold for the emergence of life as it has potential for cooperative interactions at two levels: (a) a colloid component, whose spins (exchange-coupled in MNP lattice) constitute the particle’s composite spin, and (b) the dipolar interactions between the components themselves. Indeed, anisotropic dipolar interactions in fluids impinge on fundamentals, such as direction dependence, intrinsic long-range nature and susceptibility to external forces (Wei and Patey, 1992; Weis et al., 1992; see Klapp, 2005 plus references; see Field-Controlled Scaffold Organization), and are biologically intriguing (Tavares et al., 1999). In ferrofluids (single-domain MNP suspensions in carrier liquids), these can lead to correlations between neighboring dipoles in growing fractal clusters, wherein to minimize dipolar energy, dipoles prefer to be parallel head-to-tail or antiparallel side-to-side (Pastor-Satorras and Rubí, 1995, 1998). In zero-field (for particles with large magnetic moments) dipolar interactions can lead to isotropic fractal aggregates, qualifying them as SOC systems (no external driving). Thus the expected field-induced scaling behavior was described as the response of this fractal equilibrium system at the critical point to the small external field conjugated to its order parameter (Botet et al., 2001). Now, the change from zero-field with diffusion-limited aggregation (DLA) to a field-driven one in moderate fields is expected to reduce the fractal dimension of the reversible structures (c.f. micro-particles, Domínguez-García and Rubio, 2010). The intermediate regime suggests an access to statistical features like *scaling* on the one hand, as well as *controlled mobility* on the other, via field-control. These ingredients offer a confined biological-like (level-I) system with potential for feedback effects: its *susceptible* global-configuration – dictating function – cannot be determined from properties of its components alone, and it can *influence* the orientations/dynamics of sterically coupled organics at guest-level-II (see Towards Cooperative Transitions). Here, responsive adjustments to changing external influences (via size and magnetic moment of incoming MNPs, reactions or interactions at level-II, fluxes, etc.) can affect the network’s capacity to “function” (say transport, see Percolation of Heat, Electrons), thus providing a basis for selection of a configuration. The potential to collectively respond to external changes seems an important requirement for a hosting-scaffold (level-I) in view of the penetrating influence of the environment upon a living system whose internal state adjusts to changes in the former.

Further, the ability of far-from-equilibrium living systems to act as conduits of energy flow equips them with dynamic stability. The construction of their dynamical nano-components/subsystems calls for a scaffold-medium with reversible interactions enabling their interplay with external fluxes, thermal motion, etc. (c.f. convergence of energies, Figure 2 in Phillips and Quake, 2006). Such a *controlled* organization with reversible dynamics (Whitesides and Boncheva, 2002) – as a starting-point for a cell-like organization – seems inaccessible to a host medium with irreversible linkages such as rock pores or thermally linked inorganic gels (despite their importance for generating abiogenics, or compatibility with other magnetic/physical effects). Again, in contrast to organics randomly floating within aqueous spaces entrapped in liposomal sacs or rock pores, the suggested *flow-reactor-type scenario* enables *association only of entities* *actively coupled* with the field-controlled system, such as organics bound to mineral-particles, or those interacting with bound organics, etc. (see How Cooperativity could Complement Bottom-Up Approaches). To that end the microfluidic system by Park and Kim (2010) seems promising. Furthermore, Ranganath and Glorius (2011) draw attention to the advantages of using externally controllable super-paramagnetic particles in a range of applications – from quasi-homogeneous catalytic systems to data storage. Figure 1B depicts the idea that a field-controlled and dynamically stable inorganic modular organization (c.f. Cairns-Smith, 1985; can (i) support the gradual evolution of organic mixtures at guest-level-II, (ii) be compatible with the simultaneous emergence of different kinds of organic networks/autocatalytic subsystems (c.f. Gánti’s, 2003 three subsystems), (iii) *simultaneously affect any coupled subsystems* and thus hasten their mutual cooperative interactions, thanks to the influence of the environment on its own *d.o.f.s*, e.g., via an SOC mechanism, and (iv) by virtue of its capacity for some *primitive functions*, provide a *selection basis* toward its own “takeover” by superiorly functioning organic networks. Note that this crucial role envisaged for an inorganic functional scaffold only concerns the initial stages of life’s emergence, for providing a feedback circuit between levels I and II till both became organic-based.

To get an intuitive feel for the organizing power of a field, think of system-components as compasses detecting/responding to magnetic field lines, or iron filings showing the lines of force from a bar-magnet. Similarly liquid-dispersed MNPs form north-to-south chains, joining together end-to-end, while adjacent strings show a repelling property. In a similarly polarized ferrofluid^1^ this particle alignment effect is spread uniformly throughout the liquid medium and a sufficient field for overcoming gravity/surface-tension can make spikes appear (e.g., see Peter Terren’s website)^2^. In fact, the remarkable similarity of magnetic/electric fields on MNP/thread suspensions, respectively, to the mitotic spindle, led Rosenberg et al. (1965) to study the effect of fields on cell division and related applications. Also, the dimensions of a cell ∼10–100 μm; protein ∼5–50 nm; gene ∼2 nm wide; and 10–100 nm long (Pankhurst et al., 2003), show that MNPs have the same length scale as bio-molecules, thus making it possible to apply magnetic field-induced clustering and cell signaling using these tiny magnets as ligands (Mannix et al., 2008; see also Chen, 2008), and also enhance the potential of field-effects in origins-of-life research. The crucial role of fields in biology today underlying cooperative effects (see Ho, 1997 and references), also provides a natural motivation to look for *coherent* influences in the origins of life that could have caused *cooperative* *interactions*.

In this review, Section “Living Systems; Soc; and Life’s Emergence” considers the implications of SOC in life’s emergence, after a brief look at biological systems and SOC. Section “Liquid Crystals; Scaffold Paradigm; and Bottom-Up Approaches” and Table 1 study Cairns-Smith’s “crystal-scaffold” organization using an Liquid crystals (LC) medium, and the potential of a soft-scaffold for assisting bottom-up approaches via kinetic aspects. Toward a “boot-strapping” scenario, we briefly look at field-induced dynamical structures – with analogies to (level-I of) biosystems – in Section “Field-Controlled Scaffold Organization” plus Table 2, and see how field-control can cause confinement of particles, influence their global-configuration, and render them as carriers for transferring heat and electrons. Section “Towards Cooperative Transitions” studies how these controlled-systems could have caused cooperative transitions in organic matter. Section “Magnetic Framboids; the Mound Scenario; a Fractal Scaffold” briefly considers fractal structures and their implications for harnessing gradients, and studies the hydrothermal mound scenario with potential for forming such structures, before conclusions in Section “Conclusions and Scope.”

**Table 1:** **The importance of being liquid crystalline**.

1	Capacity to combine order and mobility underlies its crucial role in self-organization and structure formation in biology	Hamley (2010)
2	Important biopolymers, e.g., lipids, proteins, carbohydrates, and nucleic acids display liquid crystalline phases both *in vivo* and *in vitro*	Hamley (2010)
3	Like cells LCs can amplify and transmit information	Goodby et al. (2008)
4	Like cells, they can dynamically respond to a large number of external stimuli, e.g., changing chemical concentration, temperature, light, electric, magnetic fields, and other environmental changes	Demus et al. (1998)
5	Liquid crystals have potential for electron, ion, molecular transport, besides sensory, catalytic, optical properties	Kato et al. (2006)
6	Control effects: A scaffold-medium – as a “precursor” template *a la* host-level-I – can exert its influence upon its dispersed materials – *a la* guest-level-II (see text)	Bisoyi and Kumar (2011)
6a	Far from inducing distortions various nano-materials dispersed in LC media have been observed to enhance their physical properties	Hegmann et al. (2007)
6b	The anisotropic nature and tenability of LC media can facilitate the alignment and self-assembly of nano-materials *randomly* dispersed within	Kumar (2007), Hegmann et al. (2007)
6c	Thanks to the sensitivity of LC media to small external stimuli, the latter can thereby influence the dispersed materials that are *sterically coupled* *to the host’s dynamics*	Bisoyi and Kumar (2011)

**Table 2:** **Field-controlled colloids for a “scaffold-organization” *a la* Cairns-Smith**.

	Field-control assisted “function”	Living system like characteristics	Speculation based on theory/reference/s
1a	Field-controlled aggregates (*c.f. mineral layer sequences in crystal-organization* Cairns-Smith, 1982). MNP-network configuration susceptible to external influences: size of incoming MNPs, fluxes, H-field, hosted reactions (could change local temperature or MNP’s redox state, thus its magnetic moment, etc.); these could impact transport (see Percolation of Heat, Electrons)	Confined, environment-susceptible organization; distributed control on independent interacting units; global dynamics irreducible to lower-level components, yet constrained by feedback; closely spaced configurations (c.f. Anderson, 1983); heterogeneity for reaction-diffusion patterns	Botet et al. (2001), Chantrell et al. (1982), Klokkenburg et al. (2006), Li et al. (2007); Pastor-Satorras and Rubí (2000), Richardi et al. (2008), Rosensweig (1997); see Klapp (2005), Section “Field-Controlled Scaffold Organization,” and references
1b	Coherent fields (H-field, light, electric field) for alignment, confinement of MNPs into cooperative network; resemble second-order phase-transitions	Dissipating homogeneous energy sources (ATP) to order components into cooperative organization	Taketomi (2011), Köhler and Hoffmann (2003), Riley et al. (2002), Duan and Luo (2001)
2	Close-to-equilibrium: (i) Weak, reversible dipolar interactions ∼*k_B_*T, *sustain* organization in space and time	(i) Like weak complementary-binding sustains organization in space and replicator in time	Component-level: exchange-coupling in particle-lattice
	(ii) External fluctuations can be harnessed at component as well as at system level	(ii) Fluctuations harnessed by components (work-cycles), and evolving system	System level: dipolar-coupling force
2a	Diffusing-in MNPs aligning and expanding MNP-network	“Template”-aided growth (see Diffusion Aided Processes)	Speculation for open system
	Directed transport, e.g., nucleotide oligomer-bound MNPs on garnet film)	Ratchet-dynamics of molecular motors (see Diffusion Aided Processes)	Tierno et al. (2008), Tierno et al. (2010)
b	Magneto-structural transitions (like first – order) in particle components	*Component-level:* as in work-cycles of enzymes, motors	de Lacheisserie et al. (2005), see magnetic materials (see Magneto-Structural Transitions)
c	Associative network (*c.f. varying crystal sequences* Cairns-Smith, 1982) in response to external changes.	*System level:* susceptibility to “environment”/*evolution*/analog memory.	Hopfield (1982), Huke and Lücke (2004), Palm and Korenivski (2009)
3	Potential for kinetic assistance in reactions plus trimmed phase-space of bound reactants limits possible reactions (*c.f. “side activity” in crystal paradigm* Cairns-Smith, 2008)	Flexible “templates” help juxtaposition of reactants Like flow-reactor trimming phase-space of bound reactants, curtailing side reactions	c.f. Baudry et al. (2006) See Introduction; c.f. Park and Kim (2010)
4	Far-from-equilibrium: dynamical structures via alternating H-fields/non-equilibrium conditions. Potential magnon-mode for energy propagation (*c.f. phonons in crystal lattice* (Cairns-Smith, 2008)	New self-organized structures like swimmers, self-healing structures, and others not seen in a static field. Field-tunable dispersions can store optical energy (like homogeneous ATP)	Grzybowski et al. (2000, 2009), Osterman et al. (2009), Snezhko (2011), Dreyfus et al. (2005) Patel and Mehta (2011)
5	Transfer of heat through aligned aggregate	Long-range energy transfer	Philip et al. (2008); Shima et al. (2009)
6a	Transfer of electrons (spin-polarized) through aggregate	Long-range electron tunneling	Pu et al. (2007)
6b	*Field-aligned* aggregate for spin-transmission (above)	*Chiral* assemblies for selective spin-transmission	Naaman and Zager (2011)
	Magneto-optical properties: field-induced birefringence; Faraday rotation, ellipticity; linear, circular dichroism	Analogous to properties of biological matter	Davies and Llewellyn (1980)
6c	Current carrying particle *a la* homopolar motor, Section Percolation of Heat, Electrons	Vectorial proton-transfer for torque in rotary motor	Due to Lorentz force.
7	Effect of H/electric fields on MNP/thread suspensions	Resemblance to mitotic spindle	See Rosenberg et al. (1965)
8	Merger of magnetic assemblies from different locales	Horizontal information/gene transfer	–

## Living Systems; SOC; and Life’s Emergence

### Living systems and SOC

Biological systems are self-organizing systems with a globally coherent pattern emerging spontaneously, thanks to the cooperative local interactions of its components. Important universal facets include: (1) *distributed* *control*, with all elements functioning as independent units in parallel, e.g., heterarchy in an ant colony (Dréo and Siarry, 2004); (2) controlled work-cycles of nano-machine *components*; for example, motors require a slow input from a non-equilibrium source (homogeneous) plus rectified thermal fluctuations, thanks to the *asymmetric* nature of their surfaces appropriate for Ratchet-dynamics (Astumian and Derényi, 1998; Astumian and Hangii, 2002); (3) controlled global dynamics of the *system* undergoing slow and adaptive alterations in response to environmental fluctuations; (4) chirality and polar asymmetry of building blocks for asymmetric dynamics; and (5) fractal (nested) nature of organization (Ho, 1997), enabling components to locally operate close-to-equilibrium (see point 2) with optimal efficiency despite staying globally far-from-equilibrium. A similar fluctuation-driven formation of order from disorder is a familiar phenomenon in equilibrium systems undergoing phase-transitions (see **Box 1**) – a typical form of spontaneous symmetry breaking. Note that potential energy is an integrated effect of interactions of specific arrangements (e.g., parallel/antiparallel spins), signifying order, unlike fluctuations that characterize disorder. And spontaneous symmetry breaking means that despite the system’s equations of motion being symmetrical, the instability in the internal chemistry of its components, causes a loss of homogeneity/symmetry to the system’s state (Anderson and Stein, 1985). Transitory self-organized patterns are also seen in turbulent thermodynamic systems far-from-equilibrium, e.g., convection but they do not match those of robust living systems that exhibit stability and control at each point of their dynamics, despite dissipating energy and creating entropy to maintain their structure (Anderson and Stein, 1985). Again, in vortices, typically macroscopic perturbations or higher-level structures do not modify the (internal) structure of the molecular components, unlike the bi-directional informational flows between different levels of bio-organization (Hartwell et al., 1999). On the other hand, the fractal patterns in DLA processes are somewhat reminiscent of *structural* complexities of their bio-counterparts, especially in the transporting role of diffusion (Witten and Sander, 1981).

Box 1 **Phase-transitions; order parameter.**

- Phase-transitions were classified by Ehrenfest as:
  (a) First order if there is a discontinuity in the first derivative of the free energy, in the form of a finite energy shift where the order parameter exhibits a discontinuous jump at the transition temperature *T* with an associated release (or absorption) of latent heat, e.g., as in crystallization.
  (b) Second order if the first derivatives of the free energy – namely the entropy and the magnetization – are continuous (no latent heat) at the critical point, but the second derivatives of the free energy – namely the specific heat as well as the magnetic susceptibility – show a discontinuity in the form of a divergence (or singularity), as in magnetization of a ferromagnet.
- It was Landau who first introduced a quantitative measure of order appearing at the phase-transition, through his definition of an “order parameter” (valid at or near equilibrium). It signifies the range over which fluctuations in one region of a system could be affected by those in another. In the case of a ferromagnet, the order parameter is magnetization (M).

The analogy to slowly evolving living systems becomes clearer for certain slowly driven non-equilibrium systems that can “self-organize” into a robust stationary state with a scale-invariant macroscopic behavior, owing to dissipative transport processes associated with a critical variable (Bak et al., 1987, 1988). This phenomenon – dubbed as SOC – shares some commonalities with the equilibrium concept of second order phase-transition (see **Box 1**), usually associated with scale-invariance, maintained by fine-tuning with a parameter like temperature (*T*). But unlike its equilibrium counterpart, the *critical state is an attractor of the dynamics in SOC* requiring a separation of time-scales between external driving and internal relaxation (see Bonachela Fajardo, 2008). Rather paradoxically, by providing a condition for toppling, the presence of a threshold offers a condition for stability. With a zero threshold, the component sites would be always in an active state, with the system perpetually undergoing avalanches involving many (interacting) sites but little stored energy. At the other extreme (infinite threshold) each site would store the energy received, without interactions or transport of energy; thus making the system undergo unitary sized avalanches. But a non-trivial threshold, plus a conservative rule for redistribution of energy, can lead to correlations between the sites, thus making for a spatially extended response to an external local perturbation. Thanks to closely spaced metastable states, the system can evolve by hopping from one to the other in response to perturbation-triggered avalanches where instantaneous relaxations involving the entire system occur (Bonachela Fajardo, 2008).

This kind of dynamics steadily goads the system toward a state in which the outgoing energy balances the incoming one on average, leading to a scale-free behavior. Unfortunately its meaning remains restricted, by limited consensus (see Turcotte, 2001; Halley and Winkler, 2008), to the sand-pile model (Bak et al., 1987, 1988) whose principal feature is that the (last) “fractal pile” – symbolizing the critical state – gets upset by even the addition of an extra grain of sand on top of it due to the local slope of the pile crossing a threshold. This can lead to the toppling of only two grains to an avalanche affecting the entire pile surface with sand-loss at the boundaries, thereby maintaining the stationary critical state (Adami, 1995; Dickman et al., 2000; Bonachela Fajardo, 2008). To generalize to similar phenomena for greater universality, explanations for such “unguided” critical dynamics have been proposed via their implicit association with a tuning parameter (Sornette et al., 1995; Dickman et al., 1998) like in equilibrium critical phenomena. In an absorbing-state (AS) phase-transition, a tuning parameter – the particle density – determines whether the system is in an active phase (changing in time) or in an inactive phase (stuck in one configuration). The order parameter of these transitions is the density of sites about to topple, called the activity (Dickman et al., 1998). *The coupling between order and control parameters helps attract the latter to its critical value* and brings about the phase-transition, as well as shows the possibility of a role-reversal (Sornette et al., 1995). This makes SOC a plausible candidate among scenarios for long-range correlations underlying complex bio-systems (c.f. Anderson’s, 1983 spin-glass model). This is since the susceptibility of the organism as a whole (changes in functional patterns manifest in nucleic acid sequence space) to the environment *controlling* its evolution, betrays an intrinsic *memory* mechanism, enabling it to *sense* and *respond* to its *external conditions* by changing its *internal configuration* – via an analogous coupling of control and order parameters. To that end it uses a *diversity of* *closely spaced* (metastable) *states*, resulting from *cooperative interactions* between *many* *d.o.f.s* – all typical ingredients of SOC.

### Implications of SOC in life’s emergence

Next consider a similar control/order parameter coupling-scenario between an environment and its system to understand evolution by natural selection as well as life’s emergence. Indeed, for insights into the major transitions in evolution (Maynard Smith and Szathmáry, 1995), leading for instance to improved functionality in an organism, another study (Suki, 2012) proposes that phase-transitions in the network structure associated with that function can facilitate the transition to improved functions.

Now, computer simulations have provided numerous insights (Kauffman, 1993; Kauffman et al., 2004; see Gershenson, 2012) into the ramifications of lower-scale network parameters on the global dynamics (robustness, evolvability, adaptability). And for insights into complex bio-processes, wherein higher-level behavior results from interactions at the lower-level, and which cannot be predicted from the latter’s (unit/sub-process) details, it is worthwhile to study systems comprising *non-linearly interacting entities*, i.e., whose state depends on their mutual interactions. Thus, focusing on the nature (inhibiting/activating) of interactions between lower-level units, as well as the network topology, makes functional bio-networks appear as computing/task-performing devices. Also, network features like modularity, redundancy, and scale-free topology can help the system exploit noise – an asset for functioning in a robust manner despite fluctuations (Fernández and Solé, 2006). Furthermore, natural selection may well have exploited such methods to guide the self-organization of genetic regulatory networks toward the critical regime (Gershenson, 2012). But this also brings up the intriguing possibility that such networks had themselves emerged via similar tinkering of precedent ones – in a continuous gradual process. More explicitly, we ask if the computing power of organisms that is inherent in the adaptive process (Hartwell et al., 1999) could be extrapolated backward to a rudimentary information-processing system in the pre-biotic era that may have guided the evolution of random chemical networks. Indeed Cairns-Smith’s (2008) abstraction of control-organization from these computing systems frees them from the material details and helps to extrapolate the LUCA back in time. Here, starting from the pre-biotic era, transitions (c.f. Suki, 2012) between information-processing machinery by changing materials/architecture/mechanisms, – in response to environment fluctuations – require functions associated with the ancestor to be fulfilled by its replacements.

## Liquid Crystals; Scaffold Paradigm; and Bottom-Up Approaches

### LC medium as a scaffold-organization

Complex bio-molecules – important components of the control-network – are capable of *large response-effects* *a la* de Gennes (2005), typical of soft-matter, thanks to correlated motions of their constituent atoms. They display liquid crystalline phases both *in vivo* and *in vitro*. The relevance of an LC medium to biology (see Table 1, adapted from Bisoyi and Kumar, 2011), owes it to a feature of cooperativity that facilitates responses to external stimuli (apart from control and stability), but one which is missing in a random mixture of its constituent building blocks (amino-acids, nucleotides, etc.). Besides its intrinsic properties, it can act as an *influential host medium* for the evolution of its embedded materials by controlling their orientation, helping assembly, and transferring its own sensitivity to external-fields due to *steric-coupling* (point 6, Table 1; see Alignment/Orientations of Mineral-Anchored Organics), and thus makes it easier to understand Cairns-Smith’s (2008) scaffold paradigm. As non-equilibrium states are stable when they act as energy carriers, in the absence of any new functional structures appearing, this medium of cooperatively acting components can offer its own (rudimentary) capacity to act as an energy conduit. Conversely, it can be dispensed with in favor of new emerging structures with superior functions. Thus such dynamic stability ensuing from cooperativity in a medium would have provided time for the *interactions between its randomly engendered materials* to lead to the *gradual* appearance of constructs of *increasingly higher specificity* and *lower connectivity* (c.f. Kauffman, 1969), that could range from structures to complex spatio-temporal patterns, capable of canalizing energy more efficiently. This gels with Langton’s (1990) emphasis on *the vital dependence of complex computations* requiring diverging correlations in time (for memory), and length (for communications), *on phase-transitions*, in the context of life’s emergence, by insisting on the primitive functions required for computation, viz., the transmission, storage, and modification of information, so that it can spontaneously emerge as an important factor in the dynamics of a system.

### The scaffold as a controlled cooperative organization

Rather than suggesting the spontaneous emergence of context-laden biological language from random processes alone, the scaffold paradigm offers a *pre-existing* environment-responsive functional inorganic *control-organization* – level-I – to host/guide the (irreversible) evolution of random organic reactions/assemblies – level-II. Conceptually, assistance from collective crystal-vibrations (Cairns-Smith, 2008) would have elevated the status of a thermodynamically motivated proto-metabolic process to that of a *function*, while gradual organic “takeover” of level-I would lead to today’s control-network (level-I) *feedback-coupled* with the metabolic-network (level-II), supplying energy and building blocks. Note that in contrast to living systems – whose ordering source comes from their dissipation of energy (closure; Shapiro, 2007), a scaffold awaiting “takeover” is not constrained to follow this pattern. But it does need a sustained source for its ordering and access to non-equilibrium sources. Now autocatalytic cycles, e.g., reverse citric-acid cycle (Morowitz et al., 2000), may have served as disequilibrium-releasing channels besides providing building blocks for the control-network (Copley et al., 2005), although they require mechanisms providing *kinetic assistance* and *pruning of side reactions*. Today, regulated enzymes lower activation energy barriers by controlling the orientations of the reactants. True, it is hard to imagine a corresponding variety of enzyme-like specifically binding surfaces via a crystalline matrix (see Orgel’s, 2000 perplexity at Wächtershäuser’s conclusion). Nevertheless, the *effect eventually caused* by the different enzymes, viz. of *trimming the phase-space of the reacting species* (level-II), could have been achieved via the association of some pre-existing control-organization – level-I – with the random pre-biotic reactions.

### Cooperativity: To complement bottom-up approaches

Approaches considering the emergence of a non-genomic replicator by random drift through autocatalytic closure of simple catalytic molecules before template-replicators (Dyson, 1982; Kauffman, 1986; Bollobas and Rasmussen, 1989; see Hordijk et al., 2010) may have overlooked such a “top-down” pre-existing kinetic principle helping its onset. Besides, a cooperative colloidal system assisting a spontaneous process (function) seems the appropriate medium for supporting/awaiting cooperative phase-transitions in random networks, and selecting gradually emerging ones “taking-over” its functions. Now, in looking for the “ultimate ancestors of modern enzymes,” Dyson indeed considers the possible role of clay crystals or iron sulfide membranes, but merely as *passively confining* surfaces, which obscures their possible impact on the probabilities of a gradual transition from a random collection of catalytic units to a *cooperative* population, say via the mean-field approximation (c.f. Curie–Weiss model of a ferromagnet), since the population of molecules slowly diffuses over the transition barrier. Nonetheless, taking inspiration from Dyson’s (1999) “cells-first” model, we explore the possibility of a *directed* way to more structured quasi-stationary states – “*possibly with active biochemical cycles and higher rates of metabolism*” – from within a random and disorganized population of molecules, “*in an assemblage of many droplets existing for a long time*.” As mentioned (see Introduction), binding to field-controlled MNPs would have caused a drastic reduction in the phase-space available to the reacting organics toward bringing about such a transition thanks to the invisibility of H-fields to organics. It is logical to suppose that magnetic-interactions would restrict the possible orientations of the organic-bound mineral-particle; this physically rules out some interactions/reactions, while kinetically assisting the feasible ones thanks to flexible magnetic “template-surfaces” (Baudry et al., 2006; Ommering, 2010; see Towards Cooperative Transitions).

As a scaffold hosting random reactions, the field-organized system of nano-particles has potential to fulfill the requirements of distributed control and kinetic assistance in top-down and bottom-up approaches, respectively, to the origins of life (c.f. Sun et al., 2007). And as the interplay of order and disorder at all scales is also feasible via magnetic d.o.f.s, the emergence of dissipative living systems (c.f. Nicolis and Prigogine, 1977) is postulated to have started from such a scaffold-organization dissipating (coherent) field energy for its formation. Although close-to-equilibrium initially, over time it got slowly pushed further and further away from equilibrium upon gradual “takeover” by (its selected) organic-based complex components, with an analogous capacity of dissipating homogeneous sources of energy for sustaining their stable and “mutually interdependent dynamics” (Cairns-Smith, 2008). This is plausible since the entropy of the super-system – the controlled system plus its environment – would then increase at a faster rate. This field-controlled system offers a mechanism for (i) confining adsorbed organics, (ii) giving access to diffusing-in “food”/materials, (iii) permitting generated “wastes” to diffuse out, hence acting like a flow-reactor with analogy to Dyson’s pre-biotic “cell.”

## Field-Controlled Scaffold Organization

From among a variety of magnetic effects having implications for life’s emergence the chief emphasis will be on reversible field-induced aggregates to simulate an evolving biosystem (see Table 2). That such aggregates can form (Taketomi, 2011) encourages the assumption of their presence in pre-biotic locales, although here one expects greater system-complexity than in the following studies, since there could have been no control on parameters (particle sizes, composition, etc.). But a chief concern is the absence of steric-effects in surface-modified synthetic ferrofluids, to avoid short-range attractive forces. This leaves unaltered action-at-a-distance effects like co-localization of particle-anchored organics, but could affect the scenario of a field-controlled scaffold. Nevertheless, the mutual interplay of magnetic-attraction and charge-repulsion – as in framboid formation (see Magnetic Framboids; the Mound Scenario; a Fractal Scaffold) – shows a way to register short-range repulsion between particles.

### Brief background

**Figure 2:**
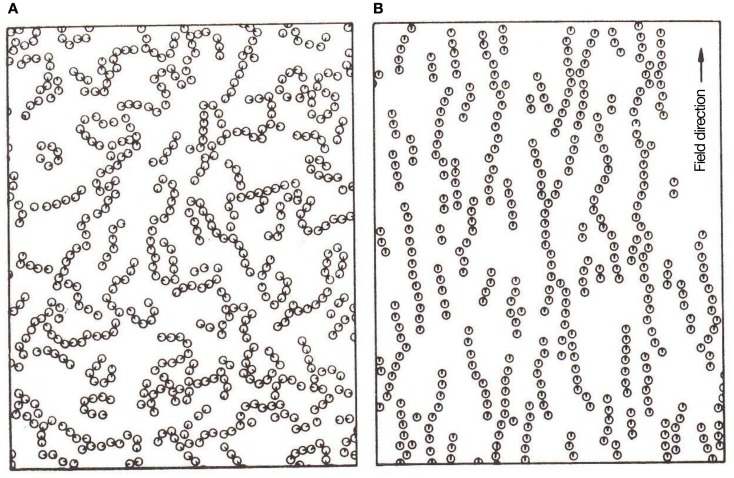
**Monte Carlos simulation in 2D: (A) clustering without H-field; (B) chaining under H-field**. Reprinted with permission from Chantrell et al., 1982. Copyright 1982, American Institute of Physics; see also Rosensweig, 1997.

Thanks to thermal fluctuations, magnetic single-domain nano-particles – key players in this scenario – are disoriented at room temperature. A moderate H-field suffices to break the rotational symmetry of such nano-particles, by imposing a directional order against their thermal fluctuations, see Figure 2, taken from Chantrell et al. (1982; see also Klokkenburg et al., 2006; Richardi et al., 2008). Li et al. (2007) describe field-induced aggregates as a phase-separation of a particle-concentrated phase from a dilute one. These (close-to-equilibrium) ordered structures – requiring about tens of milli-Tesla fields for their formation – are dissipative in nature, breaking up when the field is switched off. They are also amenable to control parameters like field strength, sweep rate, concentration, strip-width and strip-thickness. Thus, with the external H-field exceeding a critical value, the original MNPs started to agglomerate into magnetic columns and, with its further increase, formed several levels of ordered structures (Yang et al., 2003). As checked by small angle neutron scattering, chain size also depends on the strength of inter-particle-interactions (Barrett et al., 2011).

An important property of MNPs is that of anisotropy (see the classical Stoner and Wohlfarth, 1948 model); so that the applied field helps the hysteretic rotation of the magnetization to jump over the magnetic-anisotropy barrier. Next, in general, the relaxation of a single-domain nano-particle can take place via two distinct mechanisms: (1) Brownian – the individual magnetic moments, are rigidly fixed against the nano-particle’s crystal lattice so that the particle rotates as a whole; (2) Neel – the individual magnetic moments rotate within the (fixed) nano-particle. But this would also depend on its physical state. Thus, taking particles whose magnetization is not completely frozen (Neel relaxation time much faster than their measurement time), and dispersing them in a liquid medium would give the colloidal particle’s magnetization both Neel and Brownian modes of relaxation. The latter – proportional to the crystal volume – characterizes the viscous rotation of the entire particle (irrelevant for dry powders), unlike the former (an exponential function of the volume). Therefore the Brownian mode for return to equilibrium becomes the dominant process for large single-domain particles suspended in a liquid medium. Its characteristic time scale can be studied via ac susceptibility; thus an increase in hydrodynamic radius, such as upon binding to organic ligand – e.g., biotin to avidin-coated nano-particle (Chung et al., 2004) – resulted in a shift in the magnetic susceptibility peak vs. frequency curve at 210–120 Hz. Importantly, the degree of *inter-particle-interactions* (c.f. Mørup et al., 2010) can significantly affect this relaxation mode (relevant for further diffusing-in particles into an aggregate). Recall also that over and above the orienting effect of a field, a further enhancement of the magneto-viscous effect (velocity-gradient caused rotation of suspended particles, hindered by field applied perpendicular to sheer flow) was attributed to structure formation (Pop and Odenbach, 2006). Furthermore, since the dipolar interaction between two neighboring particles increases with decrease in inter-crystal distance, the particle’s aggregation-state should have an effect on the Neel relaxation, due to the dipolar inter-crystal coupling aspect of the anisotropy (Laurent et al., 2008).

### Analog for confinement

Field-induced (dipolar) interactions offer a ready mechanism for confinement of MNPs by overcoming thermal fluctuations, see Figure 2 (reproduced from Chantrell et al., 1982). The dynamical aggregates, whose components interact via weak reversible complementary dipolar forces, are analogous to living systems with *distributed control* and whose components *dissipate* *homogeneous sources* of energy. Other than such magnetic field-induced aggregation – likely a second order phase-transition (Taketomi, 2011) – magnetic dispersions can also be ordered by other coherent sources, e.g., light (Köhler and Hoffmann, 2003), and electric field (Duan and Luo, 2001; Riley et al., 2002). This expands the scope of field-control for access to scaffolds that could have been present in a variety of pre-biotic environments. Now, agglomeration of H-field-aligned nano-particles – dispersed in a fluid – leads naturally to a bottom-up assembly compliant to top-down control (see Chantrell et al., 1982; Rosensweig, 1997), wherein the spread of the aggregate is defined by the field’s zone of influence (∼inverse square law). An equilibrium state is reached when the number of particles leaving the aggregate balances those getting attached (Fang et al., 2008). Next, we suggest that in an open system, the possibility of further particles diffusing into it and aligning to the assembly “layers” would provide an analog for “replication”/growth.

### Correspondence to machine-like components

That bio-systems choose to function near the cooperative transitions of their *myriad* *different* bio-molecules also gels with “takeover” from pre-existing modules functioning primitively via collective effects. Bio-molecular machines are many-atom containing molecules whose dynamics seems to be governed by the fluctuation-dissipation theorem (FDT; Bustamante et al., 2005). Their cooperative atomic motions enable reversible switching between conformational states for work-cycles. This seems analogous to the capacity of exchange-coupled magnetic moments in an MNP lattice to change their spin orientation in response to local variations of the external H-field (via Zeeman effect).

#### Diffusion aided processes

**Figure 3:**
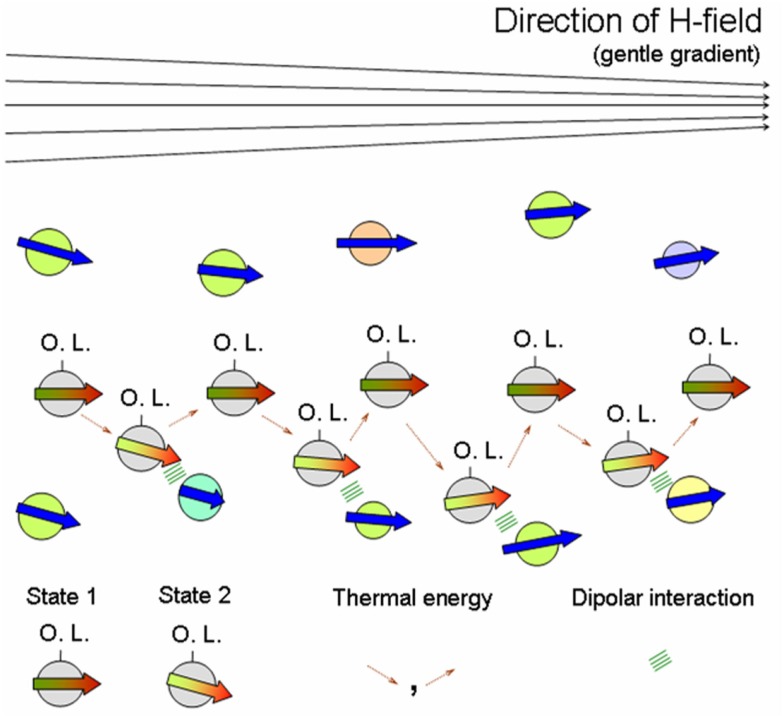
**Speculated asymmetric interactive diffusion in aqueous medium of further incoming organic ligand (O.L.)-linked MNPs – indicated by red-green arrows – through a field-induced MNP aggregate – indicated by blue arrows – in response to a gentle gradient (say, non-homogeneous rock field)**. Here particles comprising the MNP-aggregate could have differences in associated magnetic moment size (see blue arrow length), diameter, composition (see circle-colour – green, blue, yellow, pink), etc. State 1/ State 2 correspond to lower/higher template-affinity states of the diffusing O.L.-linked MNP, indicated by a grey circle having darker/brighter red-green coloured arrow, respectively. A spatially non-homogeneous H-field (direction indicated on top) provides both detailed-balance breaking non-equilibrium and asymmetry, to a diffusing magnetic dipole undergoing infinitesimal spin-alignment changes. In addition to the external field and the bath fluctuations, its orientational state is influenced by the local H-fields of its “template” partners (forming the aggregate) that would periodically perturb its directed diffusion; this magnetic interaction in State 2 is represented by green lines. Thus the dipole’s magnetic d.o.f. would enable alternating unbound and bound states, like isothermal release/attachment cycles of molecular machines on nucleic acid/protein templates, respectively. These changes would be similarly facilitated by thermal excitations from bath – indicated by dotted brown arrows – with rectification by either the gentle H-field gradient or local template-partner H-fields (Mitra-Delmotte and Mitra 2010a; see text).

Imagine further incoming MNPs, diffusing into *their* field-induced aggregate of MNPs in an aqueous medium (see Figure 2B c.f. work-cycles of a molecular motor moving on a template). Now as a dipole (associated with red-green arrow in Figure 3) diffusively migrates through the “layers” of the aggregate (indicated by associated blue arrows), in addition to the H-field and bath fluctuations, its orientational state is influenced by the local H-field of its “template” partners forming the aggregate. We also imagine a gentle H-field gradient – stemming from (inhomogeneous) magnetic rocks (Mitra-Delmotte and Mitra, 2010a) – that provides both detailed-balance-breaking non-equilibrium as well as asymmetry, to a diffusing magnetic dipole undergoing infinitesimal spin-alignment changes. The gentle gradient-driven diffusion of the migrating dipole (c.f. thermophoresis Duhr and Braun, 2006) would thus be periodically perturbed by local H-fields of its “template”-partners, leading to alternating low and high-“template”-affinity states due to the dipole’s magnetic d.o.f., rather analogous to the isothermal release/binding cycles in the priming/operative phases of the molecular machine (Schneider, 1991). Within a common FDT framework for asymmetric movements, these changes would be similarly facilitated by thermal excitations from bath, with rectification by either the gentle H-field gradient or the fields of its local “template”-partners (see Figure 3). Note also that binding to non-magnetic ligands (e.g., organics) would increase the net potential energy barrier of the particles for interacting with their “template”-partners, compared to their ligand-free counterparts. Hence, greater diffusive exploration of the organic-bound particles leads to a bio-molecular motor-like scenario, while the entrapment of the isotropically unshielded ones into an expanding network of dipolar interactions has the appearance of growth phenomena.

Now, a magnetic ratchet seems promising for the controlled directed transport of micrometer-sized colloids at the solid-liquid interface, as displayed by bio-nano-machines using the ingredients of non-equilibrium source, asymmetry, and a periodically varying potential in space/time. Tierno et al. (2008) achieved this on the surface of a ferrite garnet film with a magnetic domain pattern forming a periodic array of stripes with magnetization alternating up and down, and applying time-dependent external magnetic field pulses. Their video-microscopy tracked experiments show the transversal motion of particles on the hard film providing the local “template” fields (Tierno et al., 2010). This seems to have potential for being scaled down to nanometer-sized heterogeneities toward a magnetic shift register. Further, tunable heterogeneous field-variations on the nano-scale have not only been used for the controlled movement of aqueous phase dispersed MNPs, but also for their separation based on size of the particles (Tierno et al., 2008). The fact that the latter could be used to separate complementary oligonucleotides via a “hot zone” for melting the DNA strands, shows their compatibility with the energy-scales required for controlled bio-molecular interactions, and suggests their relevance for envisaged scaffold effects. Also, an interplay of magnetic with micro-convection (Mast and Braun, 2010) effects could have potential to cause periodic binding and de-binding between interacting particles.

Plausible mechanisms for “organic-takeover” include the autonomous motion of Janus particles whose surfaces are designed to have asymmetric chemical properties (see Baraban et al., 2012). For example, the catalytic action at one end of the particle generates an anisotropic chemical gradient across its surface, and this self-generated force drives the particle’s movement through a liquid medium. Initially during the transition, an external field’s orienting effect may well have guided such directed migration (as in Kline et al., 2005; Gregori et al., 2010) before other control mechanisms such as today’s chirality-based ones got installed.

It is important that the size scales of the non-magnetic colloids be kept in mind, when assembling bio-molecules using magnetic effects. For instance, in a magnetizable fluid, large non-magnetic colloids ∼100 nm have been shown to be pulled toward the *lower end* of the field gradient (exactly opposite to their magnetic counterparts) called negative magnetophoresis (Yellen et al., 2005; Halverson, 2008) – a method used for their manipulation and assembly by magnetic fields. This volume effect is likely to be negligible for organic ligands, like small peptides considered here; for comparison, a large 20 kDa peptide (∼170 amino-acids) has an *R*_min_ of 1.78 nm (Erickson, 2009).

#### Magneto-structural transitions

Now, secondary effects of magnetism in a substance are caused by couplings between its different physical properties: magneto-caloric, magneto-electric, magneto-optic, magneto-striction (de Lacheisserie et al., 2005), analogously to similarly coupled d.o.f.s (thermal, elastic, electric, etc.) of complex bio-molecules (Cope, 1975). This raises the possibility that similar transitions in magnetic mineral-particles (Hemberger et al., 2006) comprising field-structured aggregates had assisted some work-cycles, especially since surface-to-volume effects become sizeable at the nano-scale. For example, in the priming step in molecular machine functioning (Schneider, 1991), energy is supplied by a field-like (homogeneous) source, typically ATP, plus thermal motions captured from the bath. This is followed by the operating phase wherein dissipative ordering for information gain – recognizing a surface and reducing its conformational uncertainty – and release of entropy to the bath, takes place. The energy-shift via entropy reduction is effectively a *first-order phase-transition*. In the corresponding magnetic scenario for directed transport, an accompanying *magneto-caloric effect* can permit an interchange between system-entropy and bath temperature under isothermal conditions; also a magnetic field-controlled nano-particle assembly mimics recognition-based binding interactions between particle surfaces. Again similar to spatial field inhomogeneities causing motor-like effects, temporal field-variations can cause binding/release cycles between interacting MNPs, analogously to complementary bio-surfaces.

Note that heat released from a reaction, can alter the magnetization of the particles, vide Néel’s (1949) study. Further, two analogies of magnetic mechanisms to bio-molecular ones are intriguing: (1) the activation energy of a substrate in a chemical reaction is similar to the anisotropic energy hump of a single-domain magnetic nano-particle, flipping from one easy direction to the other; and (2) the interconnections between magnetic elastic and thermal properties in magnetic shape memory materials are rather reminiscent of enzyme dynamics. For example, a change in the material’s magnetization by changing an external H-field can not only bring about its deformation (magnetoelastic effect) but also an entropy variation (magneto-caloric); likewise a deformation due to an applied stress, can cause both a magnetization and an entropy change (Giudici, 2009; c.f. martensitic-like transformations in cylindrical protein crystals, Olson and Hartman, 1982). Alternatively, similar shape memory effects could also have been effectuated by the diffusive entry of small thermo-responsive polymers, and subsequent binding to magnetically heatable colloids in the scaffolds (Mohr et al., 2006; Schmidt, 2007; Zheng et al., 2009).

### Global evolution of aggregates

The field-induced assembly of dispersed nano-particles falls under the general category of granular systems with complex interactions (Aranson and Tsimring, 2006), with weak magnetic dipolar interactions providing a global correlation mechanism. The analogy between electric dipolar interaction-based organization in living systems and magnetic dipole interactions in a reversible aggregate (Taketomi, 2011) wherein the latter can be influenced by an externally applied H-field, makes them interesting as scaffold-systems *a la* Cairns-Smith. Ideally, a completely reversible system can capture the interplay between several competing factors, such as magnetic dipolar interactions, thermal fluctuations, screening effects of the medium (Pastor-Satorras and Rubí, 2000). Intriguingly, the complex effects of the long-range magnetic dipolar interaction (Huke and Lücke, 2004 plus references) – itself dependent on the macroscopic distribution of the particles – leads to feedback between the external and internally generated fields. This scenario seems to be analogous to the sensitivity of the internal state of living systems to external influences. Although we are unaware of experiments that correspond exactly to these speculations, nevertheless, some insights can be had from the seminal associative memory model of Hopfield extrapolating from physical systems to spontaneous bio-computation as a collective property of autonomously functioning units (Hopfield, 1982). Also, the simulations (Ban and Korenivski, 2006; Palm and Korenivski, 2009) employ a ferrofluid-based associative neural network for pattern storage, wherein inhomogeneous H-fields influence dipole–dipole interactions in the network, with the respective transition probabilities satisfying detailed-balance.

In this context, Breivik (2001) first used a life-like system of magnetic floating objects plus thermocycler, as instantiation of uncertainty reduction in producing complementary sequences, and for relating thermodynamics to information – defined as the shared entropy (via patterns) between two independent structures – in living systems. Even without catalysis, spontaneous interactions between monomers bound to a polymer resulted in complementary-string formation in response to environmental temperature fluctuations, thereby demonstrating the self-organization of template-replicating constructs toward Darwinian evolution. Although he used macroscopic objects, this scenario is down-sizeable.

### Far-from-equilibrium regime

Organic bonds (at level-II) could prevent dissociation of field-induced aggregates and enable their drift to locations providing non-equilibrium conditions (c.f. Goubault et al., 2003). For unlike static field-induced equilibrium-like clusters, alternating fields can provide interesting configurations, e.g., dynamical self-healing membranes (Osterman et al., 2009), and swimmers (Dreyfus et al., 2005). Further, spinning ferromagnetic disks at the liquid-air interface assembled into patterns due to interplay of repulsive hydrodynamic (vortex–vortex) and attractive magnetic (coupling to average field of rotating external bar-magnet) interactions (Grzybowski et al., 2000, 2009; Whitesides and Grzybowski, 2002). Again, dynamic elongated self-assembled structures – suspended at the liquid-air interface – emerged in a certain range of excitation parameters owing to competition between magnetic and hydrodynamic forces. Furthermore, self-propelled “swimmers” formed upon spontaneous symmetry breaking of the self-induced hydrodynamic flows (Snezhko, 2011).

Now, the formation of dissipative organic assemblies at level-II requires an energy source, which a scaffold with a *capacity to* *store* (coherent) energy can support. Indeed, field-tunable aggregates can store polarized (retrievable) light, its wavelength being determined via the refractive index of microcavities formed by the aligned spheres (Patel and Mehta, 2011).

### Percolation of heat, electrons

Tunable dipole–dipole interactions between MNPs – via external field strength and its orientation, etc. – can influence heat *percolation* through the network. Recent results (Philip et al., 2008; Shima et al., 2009) show a threefold enhancement of thermal conductivity of a ferrofluid over the base fluid’s, thus suggesting an efficient percolation mechanism via field-induced aggregation of 3–10 nm magnetic particles. Very large conductivity is observed with parallel fields vs. low values for the perpendicular mode. Similarly, a field-induced magnetic dipolar network, can also transport (spin-polarized tunneling) electrons (Pu et al., 2007). The possibility of percolation of heat and spin-transmission of electrons – via dipolar interactions – in a field-induced MNP-network, hosting reactions makes it interesting to consider feedback effects. A reaction at level-II could impact the MNP-network configuration at level-I, say by releasing heat and increasing local temperature or altering the redox state and thereby the magnetic moment of the hosting particle/s (at level-I; see Appendix).

Now, thermionic emission via the Richardson effect could have provided single electrons (c.f. pairs from redox reactions) to inorganic-scaffolds, which is interesting in view of the possible role of electron-bifurcation via crossed-over redox potentials in the emergence of metabolism (Nitschke and Russell, 2012). Indeed, the gradient-rich mound scenario studies geological constraints for insights into the emergence of the universally conserved proton-pump – an energy-producing vectorial process (Nitschke and Russell, 2009; Lane et al., 2010), and where far-from-equilibrium conditions can produce dynamic-cum-catalytic mineral structures (Mielke et al., 2011; see The Mound Scenario). The higher temperature inside the mound could have caused electrons (thermionic emission from alloys) to flow in the direction of the redox gradient. It is interesting to consider the electron passage through field-induced aggregates – expected to be substantial at the gradient boundary – wherein a reversibly bound particle would suffer a torque effect; this homopolar-motor-like movement may have implications as precursors of rotary motors. Table 2 lists some analogies to (level-I of) bio-systems, as reviewed in this Section.

## Towards Cooperative Transitions

In general, depletion forces (Asakura and Oosawa, 1958; Marenduzzo et al., 2006) can enable aggregation in a given location provided there is an excess above a critical concentration of interacting particles due to specific binding or anisotropic forces. Above a threshold concentration, packing (translational) entropy stemming from shape anisotropy (causing decrease in orientational at the cost of positional entropy) could help overcome rotational entropy and break orientation symmetry, thus maximizing total entropy (Onsager, 1949; Dogic and Fraden, 2005). This route could have been accessible to rod-like mineral colloids (Davidson and Gabriel, 2005; see Hamley, 2003). Indeed, mineral LCs (Lemaire et al., 2002; Gabriel and Davidson, 2003; Vroege et al., 2006; van den Pol et al., 2010) appear promising as “readily available” candidates with potential to provide a cooperative medium with sensitivity to environmental stimuli (Cairns-Smith, 2008), and this calls for a database of such minerals in pre-biotic locales. The route to LC phases has also been attempted directly from a mixture of organics using entropic forces for achieving self-assembly. Nakata et al. (2007) elegantly demonstrated the assembly of short complementary double stranded DNA into LC aggregates. The unpaired oligomers maximize their entropy via phase-separation of the rigid duplex-forming oligomers into LC droplets (minimizing their volume). As mentioned earlier (see Introduction, Liquid Crystals; Scaffold Paradigm; and Bottom-Up Approaches), to serve as effective conduits for energy flow the components of aggregates en-route to life need to bind via weak cooperative interactions (c.f. weak/reversible and transient yet specific complementary interactions enable execution of bio-functions). Now we shall briefly review some experiments to explore the potential of field-controlled aggregates to influence the phase-space of their organic guests, noting that *alignment, complementary-binding interactions, and homo-chirality* are important requirements toward decreasing the excluded volume of packed molecules as in liquid crystalline phases (Table 1).

### Alignment/orientations of mineral-anchored organics

Field-aligned particles seem equipped for the scaffold requirement of influencing their guest particles by transferring their externally induced orienting ability to their anchored organics. We imagine that in locations enriched in interacting organics (see below), transitions in abiogenic polydisperse organics to LC phases could have been aided via coupling of their orientations with those of “doping” low volume concentrations of external field-aligned ferromagnetic particles (*a la* “ferronematic” phases coined by Brochard and de Gennes, 1970), which could have increased the effective susceptibility of the fledgling organic LCs. This decrease in the effective magnetic Frederiks threshold could have led to their alignment and fractionation in the presence of weak H-fields. Moreover, recent work (Podoliak et al., 2011) suggests that although ferromagnetic particles induce a low-field response, the intrinsic diamagnetic susceptibility of the ferronematic comes to dominate its magnetic response behavior – a scenario intriguingly reminiscent of Cairns-Smith’s “organic takeover.”

### Increasing co-localization of interacting organic pairs

**Figure 4:**
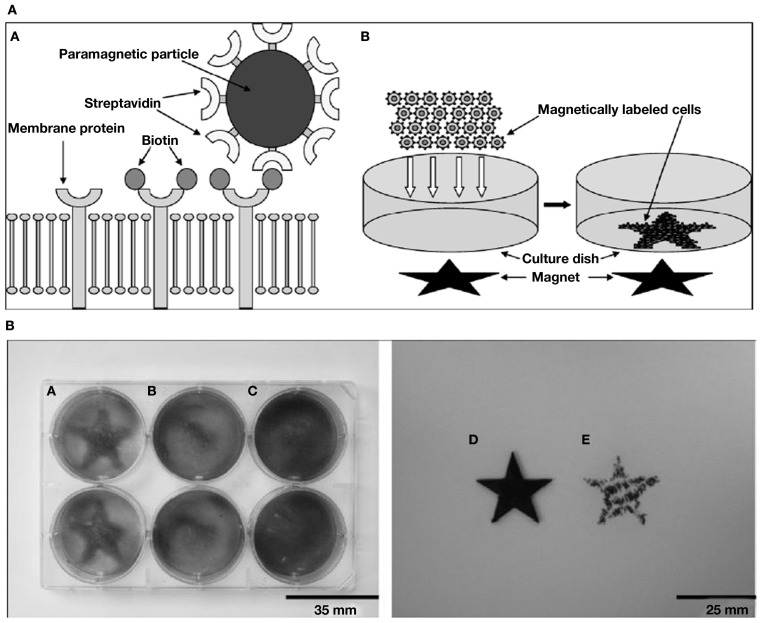
**Patterning of magnetically labeled cells by Slater and coworkers (Ho et al., 2009). (A)** Schematics of the procedure for magnetic cell labeling and patterning. A: Magnetic cell labeling. Cell membrane proteins were first biotinylated and subsequently labeled with streptavidin paramagnetic particles. B: Magnetic cell patterning. A star-shaped magnet was attached under the culture dish. Magnetically labeled cells were added and patterned onto the plate by the magnetic field. **(B)** Magnetic cell patterning of biotinylated human monocytes (HMs) labeled with streptavidin paramagnetic particles. A: Magnetically labeled HMs were successfully patterned by the star-shaped magnetic template. B: Magnetically labeled HMs were not patterned in the absence of the magnetic template. C: The non-labeled biotinylated HMs were patterned unsuccessfully by the magnetic template. D: Original magnetic template used to pattern HMs. E: Magnetic field profile of the magnetic star template used, as visualized by using iron filings to locate magnetic field maxima. Figures and legends taken from Ho et al. (2009) with kind permission from Prof. Nigel Slater; “Copyright (2009) Royal Society of Medicine Press, UK.”

As abiogenic organics were unlikely to possess shape anisotropy, *a high concentration of complementary-binding pairs with specific interactions* would have been crucial for the formation of LC phases. Indeed, for reasonable probabilities of collective transitions from disordered to ordered mutually catalytic ensembles, the ingredients required are simply stable and confined populations of molecules, whereby chance discrimination of *specific interactions* could bring about catalysis; and increasing number of such mutual interactions eventually causing catalytic reproduction of the whole set (Kauffman, 1993; Dyson, 1999). Now, Hunding et al. (2006) propose that a web of aggregates resulting from selection and growth by complementary-binding between diverse pairs of molecules across pre-biotic locales, can explain the emergence of specific interactions between like and unlike molecules in life-processes. But abiogenics could have been present in diluted solutions as well as high local concentrations via physical mechanisms (Budin and Szostak, 2010). To increase the concentration of complementary organic pairs in a given location, consider a possible magnetic d.o.f. of the organic-bound mineral-particles (see Introduction). In contrast to specific binding interactions, non-specific binding in concentrated media can be overcome by magnetic forces, thus offering a way to select pairs with binding capacity above a threshold (see Ommering, 2010). We suggest that, thanks to a field’s “action at a distance” capacity, its responsive particles – in the event of being bound to one of a pair of complementary-binding organics – have the potential to (1) aid the pairs to find each other by facilitating their detection in dilute to concentrated media (e.g., Pan et al., 2012); and (2) *chaperone* the recognition process to assist their binding (e.g., Baudry et al., 2006) thanks to flexibility of the colloidal field-aligned “templates.” Baudry et al. (2006) demonstrate how one-dimensional confinement of magnetic colloids in the presence of an H-field considerably accelerates the recognition rate between grafted receptors and their ligands, as measured by turbidometric detection of complexes in the absence of the field. They suggest that since confinement significantly augments the colliding frequency, the same also causes a large increase in the attempt frequency of recognition. An extension of such experiments by first feeding the (open) system with a slow input of nano-particles chemically conjugated to moieties like nucleotides/small peptides – and consequently checking for the incorporation of labeled complementary units – could be done in the absence/presence of an applied moderate H-field. Figures 4A,B reproduce the experiments by Slater’s group (Ho et al., 2009) who have used magnetic templates to adhere magnetically labeled cells, to illustrate how a local field, say from magnetic rocks (see Magnetic Framboids; the Mound Scenario; a Fractal Scaffold) could have influenced the dynamics of magnetic particle-anchored organics.

### Homo-chirality

Perhaps the most intriguing implication of a role of magnetic fields in life’s emergence comes from the *homo-chiral* nature of its building blocks that respond differently to left/right circularly polarized light. Indeed, the findings (Carmeli et al., 2002; Naaman and Zager, 2011) have further fueled this speculation by relating the capacity of scaled-up chiral assemblies of these building blocks to selectively transmit electrons according to their spin-polarization. To explain in slightly more detail, Rosenfeld’s (Wagnière, 2007) chiral “rotational strength” parameter **m.d** – a unique combination of P (parity), T (time-reversal) violating joint PT conservation (!) – brings out the role of the magnetic part of the e.m. field in “twisting/reorienting” the magnetic moment about the field axis. Its interaction with the electrical e.m.f. component causing electronic orbital transitions (by polarizing electron cloud across the molecule) thus leads to transitions in a chiral molecules, with d (electric) and m (magnetic) dipole components being parallel and antiparallel, respectively, hence averaging out to zero for a symmetric molecule. Feedback makes these interactions complex since oscillating H-fields can cause charges to move and vice versa. A moving charge in turn affects the properties of the carrier transporting it, and electrons have both charge and spin. Just as an electron’s response to an electric or magnetic field shows up as a translation (via charge) or rotation (via spin), likewise the response of its carrier to the electric and magnetic fields is one of charge-translation or spin-rotation, respectively. In this context recall the hypothesis of Garay et al. (1973), viz. the electron magnetic moment and the magnetic transition moment of the electronically excited chiral molecules could interact. Thus, the magnetic transition dipole could influence the probability of the triplet state of the optically active molecules, electron transport, and stereo-selectivity. Now, the findings of Naaman’s group on orienting effects of weak H-fields on bio-membranes, suggest that spin-transmission in the scaled-up versions of the chiral building blocks follow analogous rules of magnetic interaction to those of the individual building blocks. They reported *unexpectedly high* selectivity in transmission of spin-polarized electrons that are consistent with giant magneto spin selectivity in inorganic magnetic films and related colossal magneto-resistance effects. Here charge-transfer from metal substrate converted adsorbed chiral bio-molecular layers from electric to magnetic dipoles, due to *cooperative effects*. Charge redistribution leads to altered electronic structure via unpaired electrons on adsorbed molecules, rendering them paramagnetic. Although spin-filtering effects are achieved in spintronics by applying an external field to induce magnetization in ferromagnetic thin films, magnetization in their bio-counterpart, i.e., a layered assembly of dipolar chiral molecules, is based on two stages: (1) the H-field-created by transfer of charge (electron/hole) through chiral molecules aligns the magnetic dipole of the charge transferred; (2) subsequent exchange interactions in the layered domain keeps them aligned.

These observations by Naaman’s group link up two seemingly unrelated aspects of homo-chiral biological units, viz. *selective spin-transmission by their scaled-up assembled versions*. It is gratifying to note that this irreducible picture can be roughly met via H-field-aligned colloids (Pu et al., 2007; see Percolation of Heat, Electrons). Next, consider the latter as hosts of a proto-metabolic reaction: the picture of high energy electrons transferred to sinks like CO_2_ (Trefil et al., 2009) at level-II, seems consistent with that of functional “takeover” (c.f. Cairns-Smith, 2008) of the (spin-polarized) electron carriers at level-I by chiral organic assemblies, whose formation from building blocks would cause phase-space reduction. This is since chiral asymmetric structures, such as helices, provide further scope of entropic interaction-driven phase-transitions: the excluded volume decreases in going from packing parallel helices that are out-of-phase to in-phase ones, and at an angle to facilitate interpenetration into each other’s chiral grooves (Barry et al., 2006). As to the source of engendered building blocks – again in agreement with the two-level scaffold paradigm – note that field-controlled aggregates also have the potential to host the formation of chiral organic guests. Although using a different source, Rosenberg (2011) has demonstrated that substantial chiral-specific chemistry was induced by spin-polarized electrons which were provided by radiating the magnetic substrate, adsorbing the chiral organics, by an ionizing source. (The spin dependence of DOS near the Fermi energy in magnetic matter suggests how the colloids could act as spin filters). This is apart from the implications of field-controllable particles in asymmetric chemical synthesis (Ranganath and Glorius, 2011), and controlling chemical reactivity via spins (Buchachenko, 2000).

## Magnetic Framboids; the Mound Scenario; a Fractal Scaffold

### Framboids and fractal framboids

**Figure 5:**
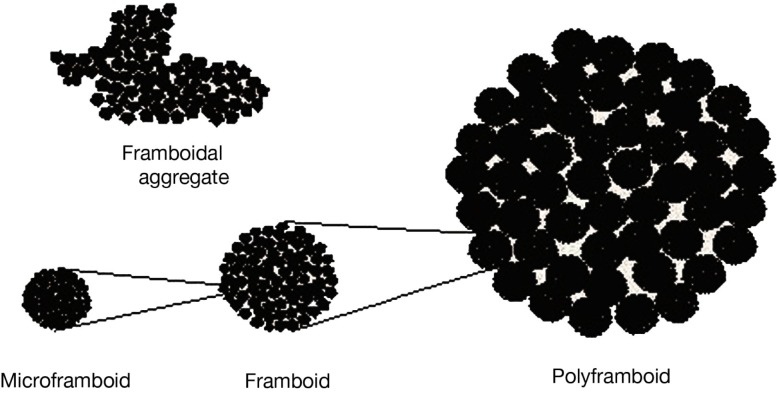
**Three size-scales observed in framboids (Adapted from Sawlowicz, 2000 with permission, see text)**. As a contrast, the top left shows a framboidal aggregate without sufficient surface minimizing forces.

As a possible scenario toward realizing a field-controlled scaffold, we briefly look at framboids, whose raspberry-patterns inspire their nomenclature. A number of structurally different minerals other than pyrite, i.e., copper and zinc sulfides, greigite, magnetite, magnesioferrite, hematite, goethite, garnet, dolomite, opal, and even in phosphoric derivatives of allophone (Sawlowicz, 2000) – form framboids, suggesting *a physical mechanism of formation*. Their formation is a dynamical self-organizing process: The nucleation of a supersaturated solution by the first-formed crystal triggers the separation of many crystals of the same size. Their ordering is an outcome of the interplay of close-packing attractive (such as surface-tension, magnetic) and repulsive (e.g., electric) forces (see Sawlowicz, 2000). Next, studying their presence in sedimentary environments, Sawlowicz (1993) found framboids to be structured over a hierarchy of three size scales: microframboids, to framboids, to polyframboids (Figure 5; taken from Sawlowicz, 2000); he suggested the formation of nano-framboids, comprising microcluster aggregations (∼100 atoms), by analogy with the three-scale framboidal hierarchy. Pictures of polyframboids and aggregations of minute particles forming spherical grains (microframboids) in framboid can be seen in Sawlowicz (1993), Figure 1 (see plates K, L). Based on observations, Sawlowicz proposed a formation mechanism by which the original super-saturated gel-droplet would undergo subsequent divisions into immiscible smaller droplets; further subdivisions would depend on a number of factors (e.g., initial size, iron concentration, gel stabilization, viscosity, activity of sulfur species), wherein a key role is played by the colloid-gel phase in leading to the fractal forms. Also, the exclusion of organic compounds led to simple framboid formation via an aggregation mechanism, while in experiments with organic substance stabilized gel-droplets, fractal framboids formed by particulation.

#### Mineral “relics”

Besides having a striking resemblance to FeS clusters in ancient enzymes (see The Mound Scenario), the mineral greigite has magnetic properties. Now today’s enzymes control electron transfers in FeS clusters (Noodleman et al., 1995, 2002) exploiting their sensitivity to local micro-environment fields (organic ligand, solvent, etc.). This gels with the picture of controlling enzymes “taking-over” from functioning catalytic-cum-magnetic components of a field-controlled network. Further, from observations of (bio-mineralized) fractal greigite framboids (Preisinger and Aslanian, 2004), it seems to be compatible with a nested organization; it can also be found in the magnetosomes of many bacteria (Reitner et al., 2005; Simmons et al., 2006). Indeed, magnetic mechanisms are hardly “unfamiliar” to living systems, being present across the kingdoms, and evolved at different times (Pósfai et al., 1998; Kirschvink and Hagadorn, 2000; Winklhofer and Kirschvink, 2010).

#### Wilkin and Barnes model

Wilkin and Barnes (1997) have explained the formation/stability of micrometer sized pyrite framboids, using an interplay of negatively charged repulsive and magnetically attractive forces (in precursor greigite), where a size >100 nm would orient crystals to the weak geo-magnetic field ∼70 μT. Assuming a spherical geometry, the critical grain diameter of constituent crystallites comprising the framboid interior *d*_c_ = 2*a*, where *a* > 1, is given by *d*_c_ = (6*k*_B_*T*/μ_0_π*M*_sat_|H|)^1/3^. This result can be obtained from the inequality *W*_WB_ > *k*_B_*T* where we define *W*_WB_ ≡ μ_0_*M*_sat_V H. Here *k*_B_ is Boltzmann’s constant and μ_0_ the permeability of vacuum. When aligned parallel to the weak geo-magnetic field (∼70 μT), *d*_c_ = 0.1 μm. (Ferrimagnetic greigite has a saturation magnetization value *M*_sat_ at 298 K ranging between 110 and 130 kA/m. On the basis of microscopic observations by Hoffmann, 1992 of natural greigite crystals, single-domain particles are roughly less than a micrometer in size).

Now for an extension of this field-assembly mechanism to the nano-scale, an extrapolation using the above formula for d_c_ shows that an H-field for accreting 10 nm sized particles – as for ferrofluids – would have to be ∼1000-fold stronger than the weak geo-magnetic field. And as there was no trace of any geo-magnetic field at ∼4.1–4.2 Ga (Hazen et al., 2008), the time when Life is believed to have been already initiated (4.2–4.3 Ga; Russell and Hall, 1997, 2006), we need extra-terrestrial sources, e.g., *meteoritic matter*, for providing local H-fields (for, e.g., see The Mound Scenario; Mitra-Delmotte and Mitra, 2010a).

### The mound scenario

**Figure 6:**
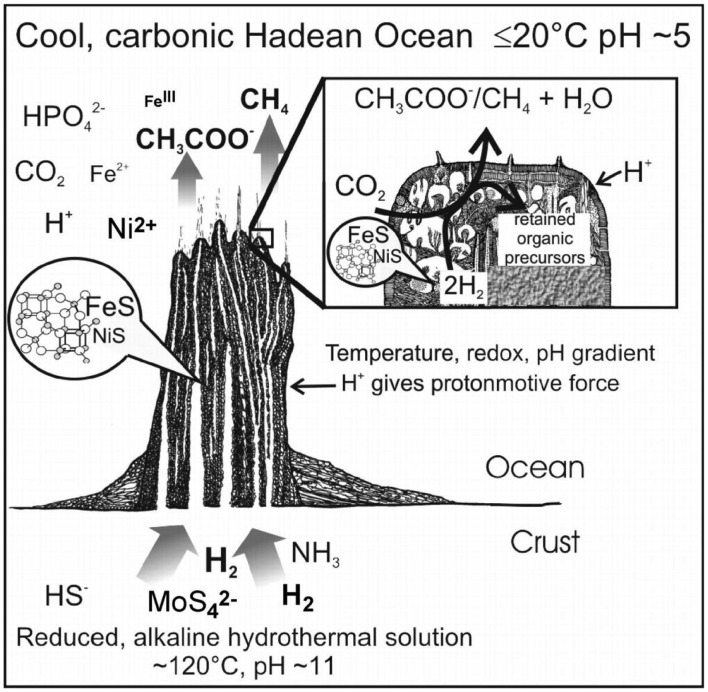
**The hydrothermal mound as an acetate and methane generator**. Steep physicochemical gradients are focused at the margin of the mound. The inset (cross section of the surface) illustrates the sites where anionic organic molecules are produced, constrained, react, and automatically organize to emerge as protolife (from Russell and Martin, 2004; Russell and Hall, 2006, with kind permission). Compartmental pore space may have been partially filled with rapidly precipitated dendrites. The walls to the pores comprised nanocrystals of iron compounds, chiefly of FeS (Wolthers et al., 2003) but including greigite, vivianite, and green rust occupying a silicate matrix. Tapping the ambient protonmotive force the pores and bubbles acted as catalytic culture chambers for organic synthesis, open to H_2_, NH_3_, ${\text{CH}}_{\text{3}}^{-}$ at their base, selectively permeable and semi-conducting at their upper surface. The font size of the chemical symbols gives a qualitative indication of the concentration of the reactants.

A colloid-gel environment in the Hadean with potential for magnetically formed framboids (Mielke et al., 2011) is the alkaline seepage site mound scenario (Russell et al., 1994; see Percolation of Heat, Electrons), wherein greigite (Fe_3_S_4_) provides the “continuity” link to iron-sulfur clusters (see Mineral “relics”). Briefly (see Figure 6 reproduced from Russell and Martin, 2004; Russell and Hall, 2006), water percolating down through cracks in the hot ocean crusts would react exothermically with ferrous iron minerals, and return in convective updrafts infused with H_2_, NH_3_, HCOO^−^, HS^−^, ${\text{CH}}_{\text{3}}^{-}$; this fluid (pH ∼ 10, ≤120°C) would exhale into CO_2_, Fe^2+^ bearing ocean waters (pH ∼ 5.5, ≤20°C), and create porous mounds consisting of brucite, Mg-rich clays, carbonates, Fe-Ni sulfide, and green rust – self-restoring reactors for titrating the hydrothermal fluid with the sea-water (Russell and Arndt, 2005) – toward reducing CO_2_ (Russell et al., 2005). Despite the low levels of bisulphide in alkaline solutions, Mielke et al. (2010) have shown the potential of the hydrothermal solution to dissolve sulfhydryl ions from sulfides in the crust that are expected to flow over ∼30,000 years – fulfilling the continuity of conditions required for abiogenesis. Here, the ensuing super-saturation in response to gradients (stark contrast of pH, temperature, etc.) would spontaneously result in colloidal precipitates of FeS (amongst other compounds, e.g., traces of W, Mo); these barriers would obstruct further mixing of the solutions, leading to the creation of non-equilibrium gradients (pH, redox, temperature; see Far-From-Equilibrium Regime–Percolation of Heat, Electrons) across these catalytic membranes, growing by hydrothermal inflation. And, abiogenic molecules (corresponding to metabolic/control levels) would coordinate with each other (Milner-White and Russell, 2010, 2011) in inorganic compartments and dynamically ordered framboidal reaction sacs (Russell et al., 1989).

**Figure 7:**
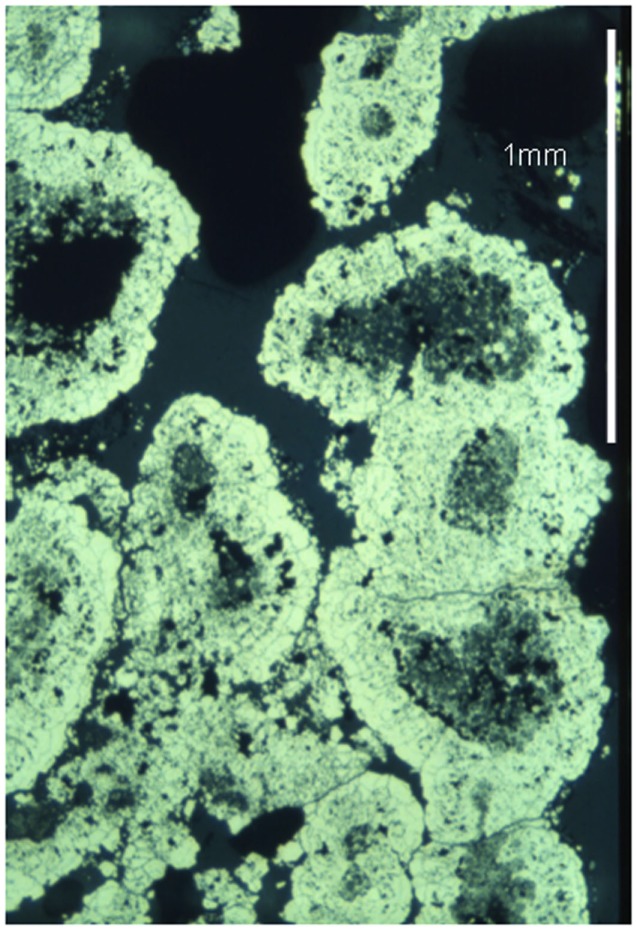
**Framboids in chimney: Sheaf system, formed from coalescing rods of anastamosing microcrystalline pyrite**. Black areas (in reflected ore microscopy of transverse section) are empty spaces; central regions are framboidal pyrite with an exterior of crystalline pyrite (picture by Dr. Adrian Boyce reproduced with his kind permission; source: Boyce et al., 1983; Boyce, 1990; see Mitra-Delmotte and Mitra, 2010b).

Indeed, spherical, ordered aggregates of framboidal pyrite (∼5 μm diameter) were found in fossil hydrothermal chimneys (Boyce et al., 1983; Larter et al., 1981; see Figure 7 provided by Boyce, 1990). Further, Russell et al. (1990) have noted the size similarities between magnetosome crystals and pyrite crystallites (∼100 nm in diameter) comprising the interior of framboids that seemed to have grown inorganically from the spherical shells of iron sulfide gel. And, it is gratifying to see laboratory-formed membranes under non-equilibrium conditions revealing globular clusters that comprise or are attached to, the inner walls consisting of mackinawite and greigite (Mielke et al., 2011). These clusters (∼1–10 μm diameter) resembling framboids, appeared similar to those in the fossilized chimneys, while the outermost crystalline layers were primarily composed of ferrous hydroxide [Fe(OH)_2_] with an admixture of nanocrystalline mackinawite; the latter were located where the highly alkaline flow could have intercepted the ferrous iron-bearing fluid, and the former where the acidulous iron-bearing solutions could access the alkaline interior of the chimneys walls with concomitant precipitation of the framboids.

#### Extension of mound scenario

Note that negatively charged mineral greigite forming under mound conditions, where pH is well above 3 (Wilkin and Barnes, 1997), resembles an aqueous-based ferrofluid. Significantly, the key to stabilizing its colloidal-gel state lies with organics (Rickard et al., 2001). The formation of colloidal magnetic minerals like greigite in the mound scenario makes it relevant to look for a control mechanism via an H-field, such as provided by rocks at the base of the mound. Primary magnetism is plausible via extra-terrestrial meteoritic particles ((Late) Ostro and Russell, unpublished work; see Mitra-Delmotte and Mitra, 2010a). And, this is expected to be reinforced by secondary magnetism thanks to serpentinization and production of magnetite. Magnetic networks can also bring together mechanisms harnessing different gradients via further colloidal/mineral precipitates enveloping the mound (c.f. H-field-influenced growth pattern of precipitated tubular structures, Stone and Goldstein, 2004).

We saw (above) that the formation of precipitates leads to progressive growth of the chimneys: their growing front is soft and gel-like, whereas the chimney parts lower down harden as a result of aging. The progressive precipitation of colloidal particles containing magnetic components could have led to detrital remanent magnetism in the chimneys, thanks to the magnetic rock-field at the base of the mound, causing the physical alignment of the magnetic particles at the time of deposition. Thus chimneys/dendrites comprising magnetic minerals, and growing as a result of slower diffusion aided processes, suggest that further magnetic ramifications such as spin-effects may have occurred within the thermal gels at the soft growing chimney front. Also, fractal aggregates – dendrites, framboids, etc. – show the possibility of reduction to lower size scales, and of being controlled by external fields (Botet et al., 2001; c.f. electric field, Tan et al., 2000).

### Fractal-network: Inorganic scaffold

The influence of network topology on its properties has attracted interest (Albert et al., 2000; Amaral et al., 2000; Aldana and Cluzel, 2003) – e.g., robustness via scale-free networks, fast communication through small world networks, etc. – although no consensus exists on its relationship to biology (Khanin and Wit, 2006). While ubiquitous fractal patterns in biology at the controlling level-I are likely to be the fruits of evolution and selection, in life’s origins such *nested* *architecture* could have been accessed via (host) inorganic-scaffolds assisting/controlling guest-level-II processes. Fractals (West and Goldberger, 1987) have been noted for their capacity for “*Fitting nearly infinite networks into finite spaces*” (Onaral and Cammarota, 2000). Indeed, a nested organization in bio-systems permits processes to operate locally at equilibrium despite the whole system/subsystem maintaining itself far-from-equilibrium (Ho, 1997). Further, these dynamical patterns are realized via reversible gel-sol transitions, using the capacity of living systems to exist at the boundary of solid and liquid states (Trevors and Pollack, 2005). Since field-induced (dipolar) ordering offers an interaction mechanism that does not make use of any chemical or geometrical constraints of the particles, we speculate that this would enable the independently acting components to explore structural configurations at every scale. And, inspired by the observations of Russell et al. (1989, 1990), Sawlowicz (1993, 2000), and Preisinger and Aslanian (2004), we have conjectured that moderate local magnetic fields could cause nested formations at the nano-scale as soft scaffolds for life’s emergence (see Merali, 2007; Mitra and Mitra-Delmotte, 2011; Mitra-Delmotte and Mitra, 2010a,b, 2012). Further, in gradient-rich (redox, pH, temperature) environments, as in the mound, gradient-dissipating organic fractal structures (Seely and MacKlem, 2012) – assembling from building blocks at level-II – could have gradually replaced the functioning modules of the control-level-I inorganic networks. The tunability of inter-particle distances in the colloidal networks (above) via an H-field (and influencing percolation of heat and electrons; see Percolation of Heat, Electrons), also suggests a route for modulating the *connectivity* of organic networks (Kauffman, 1993), the former providing an underlying manifold for guiding (c.f. Gershenson, 2012) the organization of the latter. Furthermore, a heterogeneous inorganic organization can assist reaction-diffusion patterns (Turing, 1952; Kopelman, 1989; Russell et al., 1994).

## Conclusions and Scope

Liquid crystals assemblies can be regarded as the minimal units of living systems sharing their environment-response behavior that can be traced to cooperative interactions. Next, a simplified two-tier projection of living systems shows the interdependence between the metabolic-network (level-II) and the control-network of complex bio-molecules with LC properties (level-I). Extrapolating this scenario to life’s origins, shows that macroscopic energy flow in the metabolic reaction cycles at level-II can be mapped to that in similar attractor cycles in pre-biotic locales. But no corresponding organic equivalents seem to be available for the control-network (level-I), with microscopic energy transfers, and which lower kinetic barriers and catalyze level-II reactions. To that end, Cairns-Smith’s crystal-scaffold – a level-I organization – is extended to field-responsive mineral-particles, since the intermediate regime between diffusion-limited and field-driven aggregation of anisotropic colloids seems capable of accessing the features of scaling and controlled mobility in disordered liquid medium. Such a cooperative manifold of reversible interactions achieved via coherent sources enables confinement (solid-phase-like), yet allows random sets of (MNP-bound) organics to interact (liquid-phase-like). Further, this LC-like cooperative organization is susceptible to external influences (size and magnetic moment of incoming MNPs, fluxes, etc.) that can change its function-associated configuration, leading to feedback between guest and host levels. A function – of assisting a spontaneous process – associated with an organizational “whole” corresponds to the anatomy of bio-networks, and induces selection of the functional configuration. Again, via this susceptible configuration, the inorganic network can influence the evolution (irreversible) of its sterically coupled organic guests (level-II) and cause their mutual coupling, say, via an SOC-like mechanism (among those for generating long-range correlations). We speculate that the capacity to act as a low resistance channel of energy flow would have been a pre-requisite for a long-range correlation scenario, toward becoming a computing system. Moreover its influence on the phase-space of its associated organics (see Towards Cooperative Transitions) would have oriented their assembly and dynamics toward a kinetic (Pross, 2005) direction (breaking free from thermodynamic constraints). This would have poised the system for a series of phase-transitions with appropriate replacements “taking-over” the sustenance and continuity of its functions, till achievement of closure and life’s emergence. We hope the testable ideas presented here will motivate further research.

## Conflict of Interest Statement

The authors declare that the research was conducted in the absence of any commercial or financial relationships that could be construed as a potential conflict of interest.

## Figures and Tables

**Figure A1:**
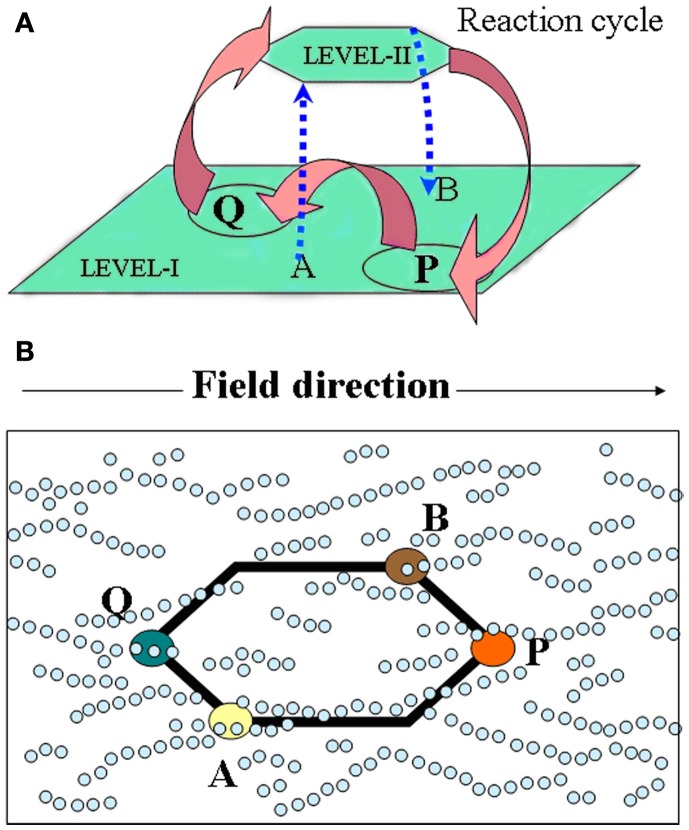
**The feedback-coupling between the control-network (level-I) and the metabolic-network (level-II), is extrapolated to the pre-biotic era to rephrase Orgel’s (2000) concerns regarding plausible assumptions on the nature of minimal information-processing capabilities of mineral surfaces for hosting/organizing a proto-metabolic cycle**. A capacity for interactions enabling long-range energy and electron transfers (represented by bold orange and dashed blue arrows, respectively) is needed at level-I – the hosting surface, depicted as a green parallelogram, – for proto-metabolic reaction cycles to organize at level-II – depicted as green hexagon. Did the host-surface at level-I have the ability to capture and channel the thermal energy released into it, say at point P (i.e., from an exothermic reaction taking place at level-II), to another spatio/temporal location, say point Q, where potential reactants (for endothermic reaction at level-II) could get recruited into the expanding cycle? Similarly electrons, required for some reactions of the cycle, would have led to exchanges (shown in blue dashed arrows) with the level-I catalytic colloids. **(B)** The possibility of percolation through an inorganic network of dipolar interactions makes it interesting to consider a field-controlled network of magnetic mineral-particles as a hosting surface to pre-biotic attractor cycles a la level-I. **(B)** Is a top view of **(A)**, where the green parallelogram representing the hosting surface is a “layer” of field-structured colloids, adapted from Figure 2, main text. We speculate that transfers of electrons and heat energy through the dipolar network (see Percolation of Heat, Electrons) could drive the magnetic system out of equilibrium. This is since each individual particle’s composite magnetic moment in turn is directly affected by its redox state, and also the local temperature, thus affecting their collective dynamics. Taken above a threshold these feedback effects have the potential to cause phase-transitions to regimes with new types of collective ordering, leading to a long-range correlation.

## Footnotes

^1^http://en.mobile.wikipedia.org/wiki/File:Ferrofluid_poles.jpg

^2^http://tesladownunder.com/Ferrofluid.htm
